# Zika, dengue and chikungunya population prevalence in Rio de Janeiro city, Brazil, and the importance of seroprevalence studies to estimate the real number of infected individuals

**DOI:** 10.1371/journal.pone.0243239

**Published:** 2020-12-17

**Authors:** André Reynaldo Santos Périssé, Reinaldo Souza-Santos, Rosemere Duarte, Fernanda Santos, Célia Regina de Andrade, Nádia Cristina Pinheiro Rodrigues, Joyce Mendes de Andrade Schramm, Edimilson Domingues da Silva, Ludmilla da Silva Viana Jacobson, Maria Cristina Ferreira Lemos, Andrea Sobral

**Affiliations:** 1 Departamento de Ciências Biológicas, Escola Nacional de Saúde Pública Sergio Arouca, Fundação Oswaldo Cruz, Rio de Janeiro, Brasil; 2 Departamento de Endemias Samuel Pessoa, Escola Nacional de Saúde Pública Sergio Arouca, Fundação Oswaldo Cruz, Rio de Janeiro, Brasil; 3 Departamento de Epidemiologia e Métodos Quantitativos, Escola Nacional de Saúde Pública Sergio Arouca, Fundação Oswaldo Cruz, Rio de Janeiro, Brasil; 4 Centro de Saúde Escola Germano Sinval Faria, Escola Nacional de Saúde Pública Sergio Arouca, Fundação Oswaldo Cruz, Rio de Janeiro, Brasil; 5 Instituto de Tecnologia em Imunobiológicos, Fundação Oswaldo Cruz, Rio de Janeiro, Brasil; 6 Departamento de Estatística, Instituto de Matemática e Estatística, Universidade Federal Fluminense, Niterói, Rio de Janeiro, Brasil; 7 Superintendência de Vigilância em Saúde, Secretaria Municipal de Saúde do Rio de Janeiro, Rio de Janeiro, Brasil; CEA, FRANCE

## Abstract

In the last 40 years, Latin America countries, including Brazil, have suffered from the emergence and reemergence of arboviruses, first Dengue (DENV) and recently Zika (ZIKV) and Chikungunya (CHIKV). All three arboviruses are currently endemic in Brazil and have caused major outbreaks in recent years. Rio de Janeiro city, host of the last Summer Olympic Games and the Football World Cup, has been specially affected by them. A surveillance system based on symptomatic reports is in place in Rio, but the true number of affected individuals is unknown due to the great number of Zika, Dengue and Chikungunya asymptomatic cases. Seroprevalence studies are more suitable to evaluate the real number of cases in a given population. We performed a populational seroprevalence survey in Rio, with recruitment of a sample of volunteers of all ages and gender from July to October 2018, within randomly selected census tracts and household. A total of 2,120 volunteers were interviewed and tested with rapid immunochromatographic test for ZIKV, DENV and CHIKV. Individuals with positive results for IgG and/or IgM from only one virus were classified accordingly, while those with test results positive for both ZIKV and DENV were classified as flaviviruses. We corrected for sample design and non-response in data analysis, and calculated point estimate prevalence and 95% confidence intervals for each virus. Arbovirus prevalence in the Rio's population (n = 6,688,927) was estimated at 48.6% [95% CI 44.8–52.4] (n = 3,254,121) for flaviviruses and at 18.0% [95% CI 14.8–21.2] (n = 1,204,765) for CHIKV. Approximately 17.0% [95% CI 14.1–20.1] (n = 1,145,674) of Rio´s population had no contact with any of the three arboviruses. The reported cases of Zika, Dengue and Chikungunya by the current surveillance system in place is insufficient to estimate their real numbers, and our data indicate that Zika seroprevalence could be at least five times and Chikungunya 45 times bigger. The high number of individuals having never been infected by any of the three arboviruses, may indicate a proper scenario for future outbreaks.

## Introduction

Brazil has been significantly affected by arbovirus epidemics since dengue virus (DENV) reintroduction in 1986 [1]. Since then, the virus spread throughout the country and the first major epidemic was described in Rio de Janeiro in 1990 [1]. In 2014, the Brazilian National Surveillance System confirmed the autochthonous transmission of another arbovirus also transmitted by *Aedes aegypti*, chikungunya virus (CHIKV) [2]. In 2015, autochthonous transmission of a third arbovirus, zika virus (ZIKV), transmitted by the same vector, was confirmed [2].

ZIKV infection has become a national epidemic in a short time, reaching 205,578 cases countrywide in 2016 [3]. During the epidemic, Brazilian health professionals reported an increased number of newborns with microcephaly, and the association between ZIKV and this disease was later proved causal [3]. By early 2020, 3,523 cases of Congenital Zika Syndrome have been reported in Brazil resulting in socioeconomic burden for the affected families [4,5]. Acute and chronic CHIKV virus disease have been related to mental health and its burden in Latin America was estimated in 25.45 disability-adjusted life-years (DALY) per 100,000 of population [6,7]. It was estimated that dengue was responsible for 1.14 million DALY in 2013 [8].

ZIKV, DENV and CHIKV (ZDC) epidemiologic studies are usually based on either surveillance of reported cases of symptomatic patients or on most affected subpopulations, such as cohort of pregnant women for ZIKV, or even on mathematical modeling approaches to estimate burden of diseases [9]. However, studies of symptomatic patients tend to underestimate the total number of cases due to the high level of asymptomatic infections [10]. Both DENV and ZIKV infections, and to a lesser extent CHIKV infection, tend to course with a high number of asymptomatic cases, but the true rates seems to vary from area to area and even inside a specific country [9,11].

After two years of high number of cases, ZIKV reports in Brazil are currently scarce and it is believed that a certain level of immunity has been acquired by its population. However, in a population of about 7 million inhabitants, only 40,431 cases have been officially reported in Rio (0.6%) [3]. Population cross-sectional designs based on serological diagnosis are able to generate more reliable data to estimate the real disease burden and for modeling approaches. However, seroprevalence studies are not common due to its high cost and complex logistic implementation, being even more uncommon in middle and low-income countries. In such countries, cross-reactivity due to co-circulation of multiple arboviruses makes the serological diagnosis more challenging, increasing the cost of such study designs [9].

Considering the co-circulation of ZDC in Rio de Janeiro city and the rarity of population seroprevalence studies to evaluate the ZDC dynamic and burden in such setting, our study aimed at estimate the real seroprevalence of all three arboviruses and possible co-infections by selecting a random sample of inhabitants geographically distributed over Rio´s territory. We used the STROBE checklist to format the article [12].

## Materials and methods

### Study design

We conducted a general populational cross-sectional study in Rio de Janeiro city, Brazil, with recruitment of a sample of volunteers, symptomatic or asymptomatic, within a randomly selected household based on the census tracts of the Brazilian Institute of Geography and Statistics (IBGE) ordered by Administrative Region (RA). Included volunteers were interviewed and tested for the presence of antibodies against ZDC between July and October 2018.

### Setting

The city of Rio de Janeiro is located in the southeastern macro region of Brazil and it is limited by the Atlantic Ocean to the south. The estimated population for 2018 was 6,688,927 (https://www.ibge.gov.br/). The climate in the city is tropical, hot and humid, with local variations due to differences in altitude, vegetation and proximity to the ocean (http://www.ipe.br/). The average annual temperature between 1981 and 2010 was 29°C, with the highest daily temperature averages (from 30° to 32°) occurring in the summer. Summertime (December to March) is also the period in which the greatest precipitations are reported (average of 205mm of precipitation in January between 1981 and 2010). The city is geographically divided into 33 Administrative Regions (AR) and 161 districts.

The study recruitment occurred during the low season risk period to acquire ZDC due to decrease of breeding sites and low vector proliferation from dry conditions, and was all based on home visits. Interviews were made during weekdays and tests and blood collection were scheduled for a second visit during weekends within 10 days from first visit so that we could include volunteers that were not at home in the first visit.

### Participants

Individuals of all ages and gender residing in permanent private households in Rio were defined as potential participants. Foreign adults living in Rio who did not speak Portuguese were not included. We also did not include adults living in collective households such as hotels and pensions, and in improvised homes.

Households were randomly selected and all residents invited to participate. However, only those who agreed to sign the consent form were included. For volunteers under 18, consent was given by legal guardians. When approaching condominium, the building manager was first contacted and the study objectives explained so that interviewers could gain access to the housing areas. Informative material specially elaborated for the study was distributed during the field activities and made available in social networks developed exclusively for the project. In addition, project researchers participated in radio and television programs to broaden the knowledge of the study in the city and facilitate the acceptance of interviewers' entry into the residences.

### Variables and data measurement

The rapid diagnostic test (RDT) kits used are based on the dual-path immunochromatographic (DPP®) platform for ZIKV, CHIKV and DENV, IgM/IgG. The RDTs were developed by Bio-Manguinhos (*Fundação Oswaldo Cruz*—Fiocruz), Brazil, in partnership with ChembioDiagnostic System INC, USA and use whole blood, serum or plasma, digital or venous puncture samples for the simultaneous detection of IgM and IgG against the three arboviruses. Results become available in 15 to 20 minutes. The test has an innovation that is a digital instrument for reading, interpreting and storing test results. Results are available in absolute numbers and in the form of categories based on pre-established cut-offs (negative, positive, indeterminate and invalid). Tests performed with human blood samples indicated sensitivity (IgM and IgG) close to 100% and specificity of 95% for IgM and 98% for IgG, but high level of cross reaction between ZIKV and DENV were reported.

Every volunteer included in the study should agree to perform the ZDC RDT using digital puncture samples. Venous blood was collected from volunteers RDT positive for ZIKV and DENV for future laboratory confirmation with plaque reduction neutralization tests (PRNT) because of the possibility of cross-reaction between the two viruses, except for children under five years of age.

All consented volunteers responded to a standardized questionnaire that was developed based on the World Health Organization (WHO) Zika seroprevalence survey document aiming at equalizing studies on the prevalence of arboviruses worldwide [13]. The questionnaire was filled in a portable electronic device (PED) to guarantee safely data storage and volunteer confidentiality. All study data were automatically and safely synchronized to a central storage and data quality control. Each volunteer received their own identification when entering the study and that was the only identification available in the electronic databases. Socio-demographic (such as age, gender and race), epidemiologic (such as housing conditions, travel to endemic areas and ways used to avoid contact with vector), and clinical data (such as current symptoms, vaccination against yellow fever and report of previous episodes of Zika, Dengue or Chikungunya fever) were collected in the questionnaire.

### Bias

We addressed the potential for selection bias by randomly appointing IBGE Rio´s census tracts and by choosing the target households equally randomly inside that area. We visited the selected areas to update the households’ information from the 2010 IBGE national census. Once selected, a household was visited at least three times before called empty. For those, a list of possible substituted target residences was available for the field interviewers. For the included household, we attempted to interview all consented dwellers. In order to accomplish it, we scheduled a second visit to the residence during the weekend so that all RDT and weekdays missed interviews could be performed.

Information bias was addressed not only by training both teams, interviewers and health professional, in the protocol, but also by performing the interviews before the RDT. Field coordinators were responsible for returning to specific households and double check the interviews. They were also rapidly available all week long to respond to any problem in the field activities such as PED and RDT malfunction.

The 17 different pairs of field investigators had at their disposal equal number of vehicles with drivers to get around the city on weekends due to the great distances to be traveled and Rio´s intense traffic. All cars were identified with the Fiocruz logo so that we could reach eventual areas dominated the drug dealers. The collected blood was stored in thermal boxes with a digital thermometer to maintain the transport temperature between 2-8°C. At the end of the activities on Saturdays and Sundays, every collected specimen was centrifuged and stored in a laboratory at Fiocruz at -70°C.

### Sample size

Rio´s census tracts from the 2010 IBGE national Census were used as primary sampling units for sample selection. The secondary units of sampling were the households, where all the residents were part of the target sample.

The sample size was guided by a prevalence of reported cases of the three arboviruses estimated at 1.5%. A minimum proportion (Pmin) of 1.5% was specified for which the relative margin of error of the estimation (dR) should be a maximum of 35%, with a confidence coefficient of 100x(1-α) of 95%. However, for a two-stage conglomerate sampling plan, we chose to consider the effects of this sampling plan on the design. We multiplied the sample size by an estimate of the effect of the sampling plan (EPA), referring to the dimensioning variable [14–16]. An arbitrary EPA of 2.15 was defined for use in the sample size calculation as there was no EPA data from previous household surveys on the subject. Considering that the average number of dwellers per household was 2.93 in the city according to the 2010 census, the household sample size was equal to 1,535 (4,500 ÷ 2.93).

$$n=\mathrm{EPA}\;x\;n_{\mathrm{AAS}}=\mathrm{EPA}\;x\;\left[{\left(Z_{\alpha /2}\right)}^{2}/d_{R}{}^{2}\right]\;x\;\left[\left(1‐P_{\min}\right)/P_{\min}\right]$$

Based on the field logistic, we defined that, in each selected census tract, we should interview residents of 10 households, leading to a sample of sectors equal to 152 (minimum number of census tract = 1511.096/10 = 151.11).

The list of census tracts was first ordered by AR and then by the average income of the households in the sector. Sectors were finally selected by systematic sampling with probabilities proportional to size (PPT), and the number of permanent private households in the sector according to the 2010 census was used as a measure of size. The prior ordering of sectors by average income within the AR, combined with the systematic selection, constitutes an implicit stratification of the sectors by income in the AR, ensuring the inclusion of households of all income levels. In each sector, the households were selected by reverse sampling [14], after the elaboration of an exhaustive list of the domiciles or update of the address book of the households of the sector.

### Quantitative variables

Our main outcomes were defined according to the RDT results. We modified a WHO recommendation for laboratory testing for Zika (https://apps.who.int/iris/bitstream/handle/10665/204671/WHO_ZIKV_LAB_16.1_eng.pdf?sequence=1) in order to define the outcomes and individuals with positive results for IgG and/or IgM from only one of the viruses were classified accordingly, while those with test results positive for both ZIKV and DENV (IgM and/or IgG) were classified as flaviviruses (FLAV) due to the possibility of cross-reactivity (FLAV = abDENV+ abZIKV+ positive sera). Coinfection was defined as having serological response to more than one virus. We analyzed as final outcomes: Dengue—DENV + DENV/CHIKV; Zika—ZIKV + ZIKV/CHIKV; Chikungunya—CHIKV + DENV/CHIKV + ZIKV/CHIK + FLAV/CHIKV; Flaviviruses—FLAV + FLAV/CHIKV. Any volunteer negative to all three studied arboviruses were classified as “No arbovirus”. All specimens classified as flavivirus are currently under laboratorial analysis with plaque reduction neutralization tests (PRNT) to differentiate serotypes of DENV and ZIKV.

Our current goal was to inform the final ZDC prevalence estimates and describe it to only few available variables already related to infection. Gender was categorized as male, female or other. Race was self-reported, and we decided to categorize in non-black (white/yellow) or black (include black and brown/mulatto race), since we did not have any native Brazilian. Age was categorized in 0 to 14, 15 to 29, 30 to 59, 60 or more years old. We used number of years in school to create the categories for literacy, and the ones used as roughly coincident with the course periods current in place in Brazil (0 to 04, 05 to 09, 10 to 12, 13 or more). We address the variable yellow fever immunization (YFI) due to the recent increase of its coverage in Rio (self-report of yellow fever immunization—yes or no). Finally, previous dengue, zika and chikungunya fever were self-reported based on sign and symptoms or diagnosis by a heath professional.

### Statistical methods

The sample was stratified and conglomerated in several stages and used procedures of disproportionate allocation of the sample. Therefore, it was necessary to calculate and use sample weights for each of the eligible interviewed residents in order to allow the estimation of the population prevalence without bias. First, basic sample weights were obtained, corresponding to the inverse of the probabilities of inclusion of the interviewed eligible residents. Then, these weights were calibrated for known population totals by gender and age group, seeking to correct any distortions in the sample distribution due to the differential non-response observed in the survey [17]. The solution adopted to correct the non-response was to model the probabilities of response by using the information available on the variables collected in the baseline survey, such as age and sex. The individuals' new adjusted weights were calculated by the ratio between their calibrated weights and the predicted values of the estimated response probabilities [18,19].

Our data analysis was both descriptive and bivariate. It is essential to incorporate the sample weights and the structure of the sample plan in the analyzes, not only in the descriptive analysis to obtain proportions and total prevalence estimates, but also in the bivariate analysis. It is also important to consider the effect of calibration on sample weights. For this purpose, we used the survey package of the R software [20].

For the final results, we used point estimates for proportions, prevalence and ratios, always associated with their confidence intervals (CI) with a 95% confidence level.

### Ethical aspects

This study is based on *Resolution n*. *466*/*2012*, issued by the Brazilian National Ethics Research Committee, and was approved in April 6^th^, 2018 (CAAE 83186318.1.0000.5240).

## Results

**Fig 1:**
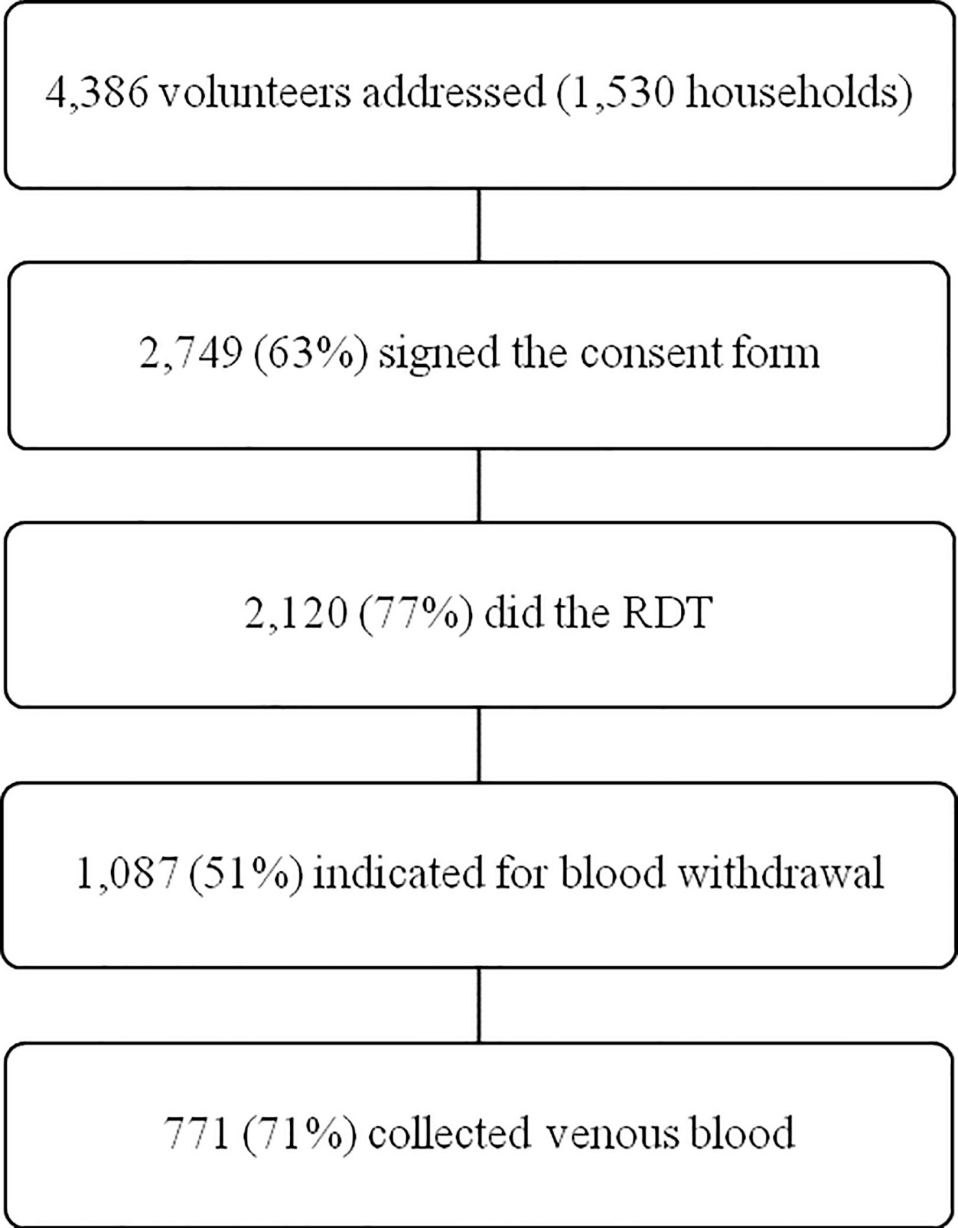
Number of volunteers at each stage of the survey.

A total of 4,386 potential volunteers were approached for study participation during the four-month period of data collection (Fig 1). Of the 2,749 volunteers (63%) who signed the informed consent, the majority were women (1,624/59%) and reported average age of 43.7 years old (SD 21,4). About 50% of the 2,120 submitted to the RDT had positive serology for both ZIKV and DENV and were eligible for whole blood withdrawn. About 70% of those eligible volunteers collected venous blood.

**Fig 2:**
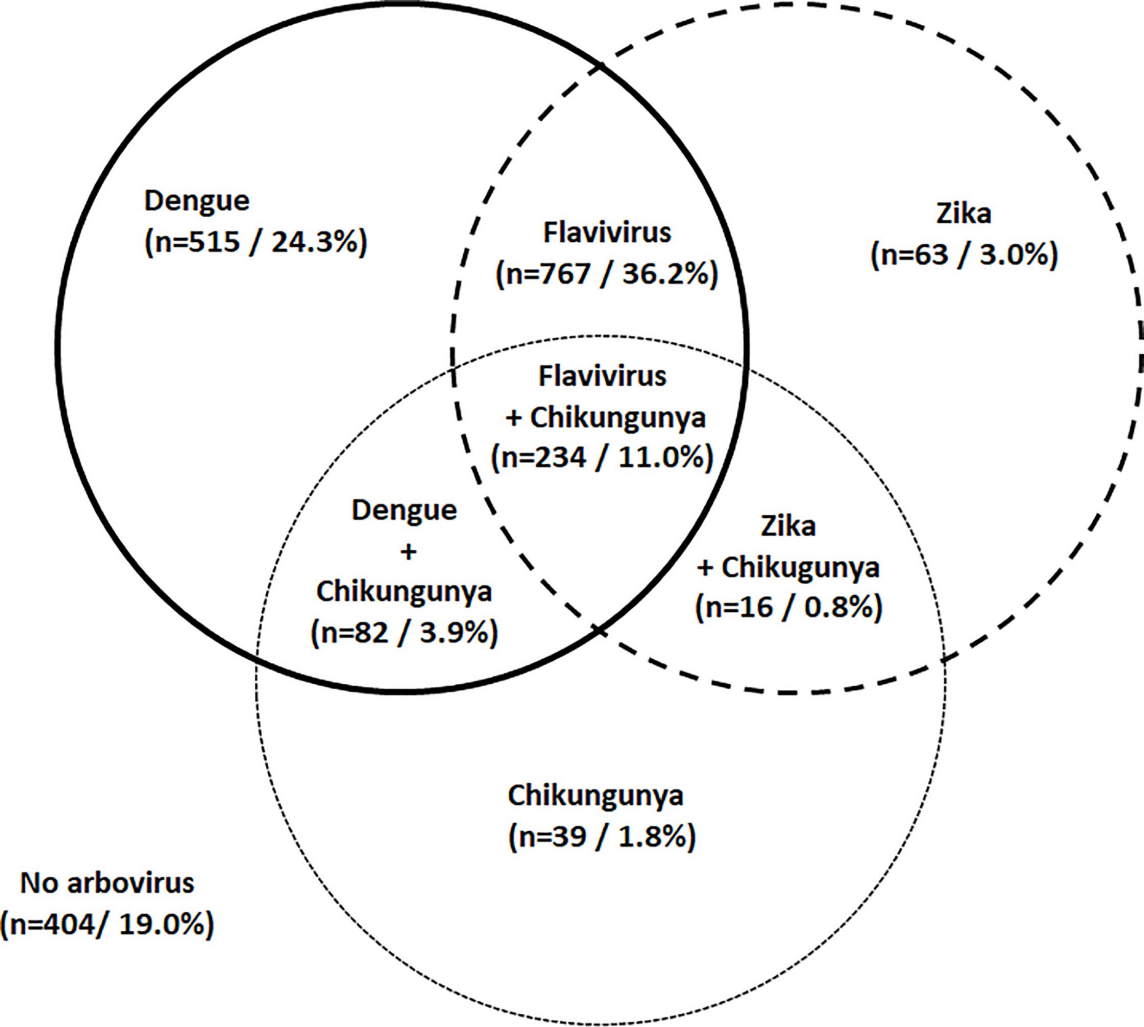
Sample distribution of arboviruses infections and coinfections.

RDT test results for 2,120 volunteers were available for data analysis. DENV RDT was confirmed in 597 volunteers, 515 (24.3%) alone and 82 (3.9%) associated with CHIKV. Most of the positive RDT were categorized as Flaviviruses (n = 1,001; 47%), 234 (11.0%) of them in association with CHIKV. Antibodies against ZIKV were found in 79 volunteers, 63 (3.0%) alone and 16 (0.8%) in association with antibodies against CHIKV. CHIKV was confirmed in 371 individuals, 39 (1.8%) alone and 332 (15.7%) in different co-infections (15.6%) (Fig 2).

Population estimates indicate that 17.1% of Rio´s population was not infected with any of the three arboviruses, while 18.0% have had contact with CHIKV. Accurate detection of DENV and ZIKV indicated prevalence of 28.9% and 3.2%, respectively, but these figures may be higher depending on the pending PRNT test results (Table 1).

**Table 1:** Population estimates of arboviruses infection in Rio de Janeiro, Brazil, 2018.

	Sample	Population Estimates		95% Confidence Interval
Arboviroses*	n	$\hat{N}$	%	
Dengue	597	1,933,085	28.9	(25.7; 32.0)
Zika	79	214,806	3.2	(2.2; 4.2)
**Chikungunya**	**371**	**1,204,765**	**18.0**	**(14.8; 21.2)**
**Flaviviruses**^**#**^	**1,001**	**3,254,121**	**48.6**	**(44.8; 52.4)**
No arbovirus	404	1,145,674	17.1	(14.1; 20.1)

*Dengue (DENV + DENV/CHIKV); Zika (ZIKV + ZIK/CHIK); Chikungunya (CHIKV + DENV/CHIKV + ZIKV/CHIK + FLAV/CHIKV); Flaviviruses (FLAV + FLAV/CHIKV)

^#^ Flaviviruses = abDENV+ abZIKV+ positive sera.

Population estimates based on sample data, indicated a predominance of women (53.1%), black race (black and brown/mulatto; 65.3%), and adults and middle age individuals (56.4% more than 30 years-old). Self-reported YFI was estimated at 55.9% (Table 2).

**Table 2:** Population estimates for socio-demographic, epidemiologic and clinical data variables.

		Sample	Population estimates		
Variables		n	$\hat{N}$	%	95% CI
Gender	Female	1,262	3,552,253	53.1	(50.1; 56.1)
	Male	858	3,139,244	46.9	(43.9; 49.9)
Race	Black	1,196	4,369,414	65.3	(61.2; 69.4)
	Non-black	924	2,322,083	34.7	(30.6; 38.8)
Age (years)	0–14	191	1,257,649	18.8	(15.7; 21.9)
	15–29	359	1,659,376	24.8	(21.5; 28.1)
	30–59	967	2,777,647	41.5	(39.1; 44.0)
	≥ 60	603	996,825	14.9	(12.6; 17.2)
Literacy (years)	0–4	123	598,759	9.0	(6.4; 12.1)
	5–9	747	2,796,182	42.0	(37.4; 46.7)
	10–12	728	2,320,560	34.8	(31.4; 38.4)
	≥ 13	508	944,375	14.2	(11.2; 17.6)
Previous YFI*	Yes	1,186	3,695,276	55.9	(51.7; 60.1)
	No	900	2,914,640	44.1	(39.9; 48.3)
PreviousDENV^#^	Yes	537	1,469,246	22.0	(19.0; 25.1)
	No	1,583	5,222,251	78.0	(74.9; 81.0)
Previous ZIKV^#^	Yes	165	564,773	8.4	(6.5; 10.7)
	No	1,955	6,126,724	91.6	(89.3; 93.5)
Previous CHIKV^#^	Yes	148	384,688	5.7	(4.4; 7.4)
	No	1,972	6,306,809	94.3	(92.6; 95.6)

* YFI: Yellow fever immunization

^#^ Previous DENV, ZIKV and CHIKV: Self-reported previous diagnosis.

Except for a higher prevalence (52.1 vs. 45.5%) and chance (OR 1.30; 95% CI 1.08–1.57) of Flavivirus among men (Tables 3 and 4, respectively), arbovirus infection seems to be equally distributed in both genders. Estimates for self-reported race, indicated that non-black race presented lower prevalence (14.8 vs. 19.8%) and chance for CHIKV (OR 0.70; 95% CI 0.51–0.97). There was no age difference for ZIKV and CHIKV, but Flavivirus prevalence increased with age from 27.2% (0–14) to 57.1% (60 or more) and RDT negative for all arboviruses was more prevalent (29.9%) for young ages (0–14). It does not seem to have a literacy gradient for seroprevalence, but DENV was less prevalent for 0 to 4 years of study and the same group presented higher figures for negative arboviruses serology (30.9%) (Table 3). Among the individuals that self-reported previous diagnosis, 166/537 (30.9%) were seropositive for DENV, 11/165 (6.7%) were seropositive for ZIKV and 120/148 (81.1%) were seropositive for CHIKV (Tables 2 and 3). Previous reported CHIKV episode was associated with a higher chance (OR 26.3; 95% CI 14.4–47.9) of a positive CHIKV serology (Tabel 4). Previous declaration of ZIKV, DENV and CHIKV was inversely associated of being negative for all tests in the no arbovirus group.

**Table 3:** Population estimates for each arbovirus according to socio-demographic, epidemiologic and clinical data characteristics.

			Dengue	Zika	Chikungunya	Flavivirus*	Arbovirus
			Positive	Positive	Positive	Positive	Negative
Gender	Female	n	359	46	**214**	**585**	251
		$\hat{N}$	1,079,821	109,068	**621,954**	**1,617,363**	651,693
		%	30.4	3.1	**17.5**	**45.5**	18.3
		IC 95%	(26.2; 34.9)	(2.0; 4.4)	**(14.4; 20.9)**	**(41.6; 49.5)**	(14.3; 22.9)
	Male	n	238	33	**157**	**416**	153
		$\hat{N}$	853,264	105,737	**582,811**	**1,636,759**	493,982
		%	27.2	3.4	**18.6**	**52.1**	15.7
		IC 95%	(23.3; 31.3)	(2.0; 5.3)	**(14.5; 23.2)**	**(47.1; 57.2)**	(12.8; 19.0)
Race	Black	n	326	54	**242**	**601**	186
		$\hat{N}$	1,226,471	158,428	**862,217**	**2,175,800**	691,273
		%	28.1	3.6	**19.7**	**49.8**	15.8
		IC 95%	(22.8; 33.8)	(2.3; 5.3)	**(16.2; 23.6)**	**(45.0; 54.6)**	(11.8; 20.4)
	Non-black	n	271	25	**129**	**400**	218
		$\hat{N}$	706,614	56,378	**342,548**	**1,078,321**	454,402
		%	30.4	2.4	**14.8**	**46.4**	19.6
		IC 95%	(26.8; 34.3)	(1.5; 3.7)	**(11.2; 18.8)**	**(41.3; 51.7)**	(16.3; 23.1)
Age (years old)	0 to 14	n	57	10	**35**	**43**	71
		$\hat{N}$	421,831	44,931	**218,980**	**342,491**	376,425
		%	33.5	3.6	**17.4**	**27.2**	29.9
		IC 95%	(18.1; 51.9)	(0.8; 9.7)	**(10.1; 26.8)**	**(16.1; 40.7)**	(18.7; 43.1)
	15 to 29	n	109	19	**70**	**164**	61
		$\hat{N}$	524,544	78,715	**302,965**	**836,319**	202,462
		%	31.6	4.7	**18.3**	**50.4**	12.2
		IC 95%	(26.5; 37)	(2.5; 8.0)	**(12.4; 25.2)**	**(43.0; 57.8)**	(7.8; 17.8)
	30 to 59	n	277	29	**157**	**489**	156
		$\hat{N}$	759,808	58,101	**483,913**	**1,505,985**	416,613
		%	27.4	2.1	**17.4**	**54.2**	15.0
		IC 95%	(24.2; 30.6)	(1.1; 3.5)	**(13.8; 21.4)**	**(49.5; 58.8)**	(12.0; 18.4)
	60 or more	n	154	21	**109**	**305**	116
		$\hat{N}$	226,902	33,058	**198,908**	**569,327**	150,174
		%	22.8	3.3	**20.0**	**57.1**	15.1
		IC 95%	(19.8; 25.9)	(2.0; 5.0)	**(16.0; 24.4)**	**(52.7; 61.4)**	(12.2; 18.2)
Literacy (years of study)	0 to 04	n	30	6	**20**	**54**	31
		$\hat{N}$	109,299	28,960	**93,920**	**245,880**	185,245
		%	18.3	4.8	**15.7**	**41.1**	30.9
		IC 95%	(6.8; 35.6)	(1.0; 13.3)	**(6.5; 29.4)**	**(28.3; 54.6)**	(16.5; 48.5)
	05 to 09	n	199	28	**157**	**386**	115
		$\hat{N}$	869,340	69,614	**573,552**	**1,356,659**	431,947
		%	31.1	2.5	**20.5**	**48.5**	15.4
		IC 95%	(25.8; 36.7)	(1.3; 4.3)	**(15.7; 25.9)**	**(42.1; 54.9)**	(10.6; 21.3)
	10 to 12	n	207	34	**136**	**371**	105.0
		$\hat{N}$	640,517	97,838	**397,389**	**1,231,597**	317,086
		%	27.6	4.2	**17.1**	**53.1**	13.7
		IC 95%	(23.0; 32.5)	(2.5; 6.5)	**(13.0; 21.8)**	**(47.3; 58.8)**	(10.4; 17.5)
	13 or more	n	158	11	**55**	**182**	150
		$\hat{N}$	308,302	18,395	**134,223**	**399,212**	206,176
		%	32.6	1.9	**14.2**	**42.3**	21.8
		IC 95%	(28.7; 36.8)	(1.0; 3.3)	**(11.0; 17.8)**	**(36.9; 47.7)**	(17.8; 26.2)
Previous yellow fever vaccination	Yes	n	342	42	**179**	**535**	252
		$\hat{N}$	1,032,753	129,848	**630,437**	**1,776,699**	706,521
		%	27.9	3.5	**17.1**	**48.1**	19.1
		IC 95%	(24.6; 31.5)	(2.3; 5.0)	**(13.0; 21.7)**	**(43.4; 52.8)**	(16.1; 22.4)
	No	n	246	35	**185**	**453**	143
		$\hat{N}$	875,089	79,635	**555,533**	**1,451,009**	417,741
		%	30.0	2.7	**19.1**	**49.8**	14.3
		IC 95%	(24.1; 36.4)	(1.6; 4.2)	**(15.1; 23.5)**	**(44.6; 54.9)**	(9.7; 20)
Previous DENV diagnosis^#^	Yes	n	166	20	**119**	**303**	40
		$\hat{N}$	412,686	45,839	**367,252**	**844,621**	146,660
		%	28.1	3.1	**25.0**	**57.5**	10.0
		IC 95%	(23.7; 32.7)	(1.7; 5.2)	**(19.5; 31.0)**	**(51.1; 63.7)**	(6.0; 15.1)
	No	n	431	59	**252**	**698**	364
		$\hat{N}$	1,520,399	168,967	**837,513**	**2,409,500**	999,015
		%	29.1	3.2	**16.0**	**46.1**	19.1
		IC 95%	(25.3; 33.1)	(2.2; 4.5)	**(13.0; 19.4)**	**(41.8; 50.5)**	(15.1; 23.7)
Previous ZIKV diagnosis^#^	Yes	n	25	11	**73**	**118**	9
		$\hat{N}$	56,006	42,908	**288,008**	**435,181**	28,189
		%	9.9	7.6	**51.0**	**77.1**	5.0
		IC 95%	(5.3; 16.3)	(2.9; 15.3)	**(37.6; 64.3)**	**(67.5; 85.0)**	(1.8; 10.6)
	No	n	572	68	**298**	**883**	395
		$\hat{N}$	1,877,079	171,898	**916,757**	**2,818,940**	1,117,485
		%	30.6	2.8	**15.0**	**46.0**	18.2
		IC 95%	(27.3; 34.1)	(2.0; 3.8)	**(12.4; 17.8)**	**(42.2; 49.8)**	(15.3; 21.4)
Previous CHIKV diagnosis^#^	Yes	n	25	4	**120**	**107**	3
		$\hat{N}$	62,771	6,571	**312,523**	**288,512**	3,974
		%	16.3	1.7	**81.2**	**75.0**	1.0
		IC 95%	(9.8; 24.6)	(0.5; 3.9)	**(71.9; 88.7)**	**(64.8; 83.6)**	(0.1; 3.6)
	No	n	572	75	**251**	**894**	401
		$\hat{N}$	1,870,314	208,235	**892,242**	**2,965,609**	1,141,701
		%	29.7	3.3	**14.1**	**47.0**	18.1
		IC 95%	(5.0; 8.9)	(4.4; 7.5)	**(0.8; 2.1)**	**(1.8; 4.1)**	(98.7; 100.0)

^#^ Previous DENV, ZIKV and CHIKV: Self-reported previous diagnosis

* Flaviviruses = abDENV+ abZIKV+ positive sera.

**Table 4:** Association of socio-demographic, epidemiologic, and clinical data with arboviruses seropositivity.

		Dengue			Zika			Chikungunya			Flaviviruses^&^			No arbovirus		
Variables		OR	95% CI	p-valor	OR	95% CI	p-valor	OR	95% CI	p-valor	OR	95% CI	p-valor	OR	95% CI	p-valor
Gender	Female	Ref	..		Ref	..	..	**Ref**	**..**	**..**	**Ref**	**..**	**..**	Ref	..	..
	Male	0.8	(0.6–1.1)	0.25	1.1	(0.6–2.0)	0.76	**1.0**	**(0.8–1.4)**	**0.61**	**1.3**	**(1.0–1.6)**	**<0.001**	0.8	(0.6–1.1)	0.27
Race	Black	Ref	..	..	Ref	..	..	**Ref**	**..**	**..**	**Ref**	**..**	**..**	Ref	..	..
	Non-black	1.1	(0.8–1.6)	0.54	0.6	(0.3–1.3)	0.21	**0.7**	**(0.5–0.9)**	**0.03**	**0.9**	**(0.7–1.1)**	**0.32**	1.3	(0.9–1.9)	0.18
Age (years)	0–14	Ref	..	..	Ref	..		**Ref**	**..**		**Ref**	**..**	**..**	Ref	..	..
	15–29	0.9	(0.5–1.6)	0.75	1.3	(0.5–3.6)	0.56	**1.0**	**(0.6–1.7)**	**0.82**	**2.7**	**(1.7–4.2)**	**<0.001**	0.3	(0.2–0.5)	<0.001
	30–59	0.7	(0.4–1.3)	0.29	0.6	(0.2–1.6)	0.29	**1.0**	**(0.7–1.5)**	**0.99**	**3.1**	**(2.0–4.9)**	**<0.001**	0.4	(0.2–0.6)	<0.001
	≥ 60	0.6	(0.3–1.0)	0.07	0.9	(0.3–2.7)	0.88	**1.2**	**(0.7–1.9)**	**0.49**	**3.5**	**(2.2–5.6)**	**<0.001**	0.4	(0.2–0.6)	<0.001
Literacy (years)	0–4	Ref	..	..	Ref	..	..	**Ref**	**..**	**..**	**Ref**	**..**	**..**	Ref	..	..
	5–9	2.0	(1.0–3.9)	0.04	0.5	(0.1–1.7)	0.26	**1.4**	**(0.7–2.6)**	**0.31**	**1.3**	**(0.9–2.1)**	**0.19**	0.4	(0.2–0.7)	<0.001
	10–12	1.7	(0.7–3.9)	0.22	0.8	(0.3–2.7)	0.81	**1.1**	**(0.5–2.3)**	**0.78**	**1.6**	**(1.0–2.6)**	**0.05**	0.3	(0.2–0.7)	<0.001
	≥ 13	2.1	(0.9–4.9)	0.07	0.4	(0.1–1.3)	0.13	**0.9**	**(0.4–1.9)**	**0.78**	**1.0**	**(0.6–1.7)**	**0.85**	0.6	(0.3–1.3)	0.12
Previous YFI*	No	Ref	..	..	Ref	..		**Ref**	**..**	**..**	**Ref**	**..**	**..**	Ref	..	..
	Yes	0.9	(0.6–1.3)	0.56	1.3	(0.7–2.3)	0.37	**0.9**	**(0.6–1.3)**	**0.49**	**0.9**	**(0.7–1.2)**	**0.59**	1.4	(0.9–2.2)	0.12
Previous DENV^#^	No	Ref	..	..	Ref	..		**Ref**	**..**	**..**	**Ref**	**..**	**..**	Ref	..	..
	Yes	0.9	(0.7–1.3)	0.74	0.9	(0.5–1.9)	0.91	**1.7**	**(1.2–2.4)**	**<0.001**	**1.6**	**(1.1–2.1)**	**<0.001**	0.5	(0.2–0.9)	0.03
Previous ZIKV^#^	No	Ref	..	..	Ref	..	..	**Ref**	**..**	**..**	**Ref**	**..**	**..**	Ref	..	..
	Yes	0.2	(0.1;0.4)	<0.001	2.8	(1.2;6.8)	0.02	**5.9**	**(3.5;9.9)**	**<0.001**	**3.9**	**(2.4;6.4)**	**<0.001**	0.2	(0.1;0.5)	<0.001
Previous CHIKV^#^	No	Ref	..	..	Ref	..	..	**Ref**	**..**	**..**	**Ref**	**..**	**..**	Ref	..	..
	Yes	0.4	(0.2;0.8)	0.01	0.5	(0.2;1.5)	0.23	**26.3**	**(14.4;47.9)**	**<0.001**	**3.4**	**(1.9;5.8)**	**<0.001**	0.05	(0.01;0.3)	<0.001

* YFV: Yellow fever immunization

^#^ Previous DENV, ZIKV and CHIKV: Self-reported previous diagnosis

^&^ Flaviviruses = abDENV+ abZIKV+ positive sera.

## Discussion

Rio de Janeiro, the second largest city in Brazil and host of the latest Summer Olympic Games in 2016 and the Football Word Cup in 2014, has been hitting hard by reemerging and emerging arbovirus epidemics in recent years. We hypothesized that most of Rio´s inhabitants have had previous contact with at least one of the three reported viruses. Our estimates indicate that more than 80% of inhabitants in Rio´s were exposed to arbovirus infection, and the most prevalent, as expected, was DENV (Prevalence = 28.9%; 95%CI 25.7–32.0) which reemerged as a public health problem in Brazil in 1990 [1]. The least prevalent among the three-target viruses was ZIKV (Prevalence = 3.2%; 95%CI 2.2–4.2), which is somewhat surprising due to the large epidemics of ZIKV in Rio in 2016 [3]. However, these numbers could reach more than 50% if all infections categorized as Flaviviruses were assumed to be ZIKV, leading to more robust estimates compared to other international seroprevalence studies [9]. Prevalence of CHIKV was estimated to be 18% (95%CI 14.8–21.2) in Rio after four years of uninterrupted report of large number of cases. Due to the circulation of different viruses from the Flavivirus genus, we were not able to rule out the DENV and ZIKV RDT cross-reactivity. Therefore, we opted to categorize this uncertainty as Flaviviruses (abDENV+abZIKV+ positive sera). Prevalence estimate for Flaviviruses reached 48.6% (95%CI 44.8–52.4). Coinfection of Flaviviruses and CHIKV was common.

Overall, DENV seroprevalence is higher in the Americas than in Asia and Africa, but rates are not homogeneous distributed in its countries [9,21–23]. ZIKV seroprevalence also varied largely and rates from 36% in the pediatric group in Nicaragua to 66% among schoolchildren in French Polynesia have been reported [24,25]. Introduction of CHIKV in the Americas is recent and seroprevalence studies are still rare [26,27]. The reported numbers (13.1–20.0%) are lower than the reports from Africa and Asia [28,29].

Prevalence estimates were slightly higher for women but gender was not found to be associated with confirmed virus serologic tests, excepted for Flaviviruses. Studies on gender-related differences are not conclusive, and while some authors indicate a higher risk for men [30,31], others indicate a higher risk for women [25,32]. Gender differences are usually explained by level of exposure to the vectors and this fact may be influenced by cultural particularities [33]. Age-group was not associated with DENV, ZIKV and CHIKV positive RDT. However, the same data for Flaviviruses indicate a gradient increase of seropositivity in the direction of older groups. All age-groups were associated with a lower chance of no serologic response to the analyzed arbovirus. These results reinforce data from other studies showing no relationship between age and seroprevalence in a new disease emergency scenario, and an age gradient in the endemic context [21,22,28]. Data on DENV, ZIKV and Flaviviruses are less reliable and further analysis will be needed once the PRNT results become available. Our non-black population tend to have lower chance for arbovirus seropositivity, and race disparity is more evident for CHIKV infection (OR 0.70; 95%CI 0.51–0.97). Although not statistically significant, the non-black population had a higher chance of serological non-response to all studied arbovirus. Our results for the literacy variable indicate not only higher prevalence for groups attending schools for less than 9 years, but also for those who attended up to 12 years. The same groups had lower chance to be negative for all studied arbovirus. Black race and low literacy may not be directly related to seroprevalence, but rather being markers of direct exposure to vectors due to lower socioeconomic status [9,21].

In a complex context of an epidemic scenario in which three arboviruses are circulating almost concurrently, sign and symptoms recall seems to be related to the intensity of complains, since Chikungunya is markedly associated to acute and chronic painful arthralgias. Those who reported previous CHIKV diagnosis based on symptoms and serologic confirmation presented a higher chance of being indeed seropositive for CHIKV (OR 26.28; 95% CI 14.40–47.95). Similar finding has been reported elsewhere [28].

Seroprevalence studies are more important to estimate diseases burden but, as in any other epidemiologic design, these results have to be interpreted with caution. We presented the final results of a ZDC cross-sectional serologic study in Rio, a major city with a large geographical territory and heavy traffic in which more than 6.5 million people live, almost 20% of them living in slams with difficult access, low cover of urban infrastructures and high rates of violence (IPP: http://www.data.rio/pages/rio-em-sntese-2). Study sample size was calculated based on all Rio´s AR and stratified by SES aiming at covering the entire territory and make our sample representative of Rio´s population. Large distances, difficult access and the constant fear of violence among Rio´s inhabitants may have had an impact on our ability to have the approached individuals sign the informed consent and test. Several strategies were used to increase recruitment and the study visibility to the Rio´s communities by using of social media, television and radio. Nevertheless, the refusal group was different in gender and age distribution and we corrected it for the final estimates by using techniques already used in national surveys. An accurate RDT to differentiate ZIKV and DENV infection was not available during the study period. Therefore, blood samples were collected for PRNT laboratory differentiation, and more than 70% of those eligible individuals accepted the procedure.

We presented a well-funded populational survey performed in four months during 2018. The observed non-response can be considered compatible with the type of research and in line with the practice of successful home surveys in Brazil [34]. The final gender and age estimates were in line with the 2010 national census and the 2015 estimates performed by the Federal Government indicating that our sample is representative of Rio´s population (https://datasus.saude.gov.br /populacao-residente/). In spite of all adversities, we were able to approach the entire target sample and recruit 63% of them, resulting in estimates likely generalizable not only to Rio´s population but also for populations with similar socioeconomic and epidemiological contexts.

Based on our estimates and the official reported numbers until the end of 2018, only a few cases of ZIKV and CHIKV infection reached health units for care, and the great majority may have had mild diseases or were asymptomatic. Our data accounted for 214,806 and 1,204,765 individuals in Rio in 2018 with serologic marker of previous ZIKV and CHIKV infection, respectively. From 2015 to December 2018, the regular surveillance system in place in Rio reported 40,431 cases of Zika and 26,810 of Chikungunya and our data indicate that Zika seroprevalence could be at least five times bigger than the reported cases to the regular surveillance system, while for Chikungunya these figures could be 45 times bigger. The reason for that could be not only the high rates of asymptomatic cases, but also difficulties for a correct diagnosis due to the little knowledge of both diseases which were only recently introduced in Rio. Moreover, the presented results also show that, even after 30 years of the first DENV epidemic and four years after ZIKV and CHIKV emergency, 1,145,674 individuals had not been infected by any of the three arboviruses, which may indicate a proper scenario for future outbreaks.

## Conclusions

Herein, we presented a more direct measurement of the immunity scenario in the complex setting of a major city in which more than one arbovirus is circulating at the same time. Our findings are important to reinforce the need for well-designed seroprevalence research in order to obtain the real burden of diseases which present moderate to high levels of asymptomatic cases. In the public health perspective, our data suggest that the current surveillance system in place is insufficient to estimate their real health and socioeconomic impacts.
